# The complete chloroplast genome of *Polygonatum cirrhifolium* (Wall.) Royle, a medicine herb

**DOI:** 10.1080/23802359.2020.1860698

**Published:** 2021-01-17

**Authors:** Pan-Hua Liao, Dong-Ling Li, Hai-Ying Tong

**Affiliations:** a Institute of Botany, Jiangsu Province and Chinese Academy of Sciences, Nanjing, China; b The Jiangsu Provincial Platform for Conservation and Utilization of Agricultural Germplasm, Nanjing, China

**Keywords:** *Polygonatum cirrhifolium* (Wall.) Royle, chloroplast genome, phylogenetic analysis

## Abstract

*Polygonatum cirrhifolium* (Wall.) Royle is a medicinal plant of commercial value. In the present study, we assembled the complete chloroplast genome of *P*. *cirrhifolium*. The total genome was a circular DNA molecule of 155,583 bp, which was made up of a large single copy region (84,412 bp), a small single copy region (18,427 bp), and a pair of inverted repeat regions (26,372 bp each). A total of 133 genes was annotated in the chloroplast genome, including 85 protein-coding genes, 40 transfer RNA (tRNA) genes, and eight ribosomal RNA (rRNA) genes. Overall, the chloroplast genome had a GC content of 37.66%. Phylogenetic analysis showed that *P*. *cirrhifolium* was closely related to *P*. *kingianum*.

*Polygonatum cirrhifolium* (Wall.) Royle is a perennial herb with high medicinal value. The rhizome of this species possesses a series of pharmacologically important secondary metabolites and hence holds vast range of pharmacological activities, such as antioxidant, demulcent, cardiotonic, energizer, hypoglycemic, antifungal and antibacterial (Saboon et al. 2016). Beside its medicinal value, *P. cirrhifolium* contains major food constituents (protein, carbohydrates, and fat) and is used for functional food (Sharma et al. 2014). Because of the great market potential, *P. cirrhifolium* is being overexploited from its wild habitats, making it become a highly endangered species (Lohani et al. 2011). A comprehensive genomic resource would help the development of conservation strategies for threatened plants (Hou et al. 2018). To promote the conservation of *P. cirrhifolium*, we assembled its complete chloroplast genome in this research.

Fresh leaves of *P. cirrhifolium* were sampled from Nanjing Botanical Garden (Nanjing, China 118°49′41.32″E, 32°3′22.74″N), and stored at −80 °C until subsequent use. The voucher specimen was stored at Herbarium of Institute of Botany, Jiangsu Province and Chinese Academic of Sciences (voucher: Liao20200708-1). Genomic DNA extracting was done by using a DNeasy Plant Mini Kit (Qiagen, Valencia, CA). A paired end library with an insert size around 350 bp was constructed and sequenced on the Illumina NovaSeq system (Illumina, San Diego, CA). Following sequencing, a total of 5.82 Gb of raw data (38.80 M reads) were generated (NCBI Sequence Read Archive accession number SRR12778006). Raw reads were quality filtered through Trimmomatic v0.32 (Bolger et al. 2014). After filtering the raw reads, clean reads were mapped to the reference genome by NOVOPlasty (Dierckxsens et al. 2017). Initial gene annotation was done by Geneious R11 v11.0.5 (Biomatters Ltd, Auckland, New Zealand) based on the chloroplast genome of *P. kingianum* (MN934979; Jin et al. 2020). Genes that could not detected were further identified by Blastn (https://blast.ncbi.nlm.nih.gov/Blast.cgi). The annotated cp genome was deposited in GenBank under the accession number MT955358.

The cp genome of *P*. *cirrhifolium* presented a circular double-stranded DNA structure of 155,583 bp, which consisted of two inverted repeat (IR) regions of 26,372 bp each, a large single-copy (LSC) of 84,412 bp, and a small single-copy (SSC) of 18,427 bp. The *P. cirrhifolium* cp genome was predicated to contain 133 genes, including 85 protein-coding genes, 40 tRNA genes, and 8 rRNA genes. For the annotated genes, seven protein-coding genes, eight tRNA genes, and four rRNA genes were duplicated in the IR regions. A total of 15 different intron-containing genes were detected, with 13 contained one intron and two contained two introns. The overall GC content of *P*. *cirrhifolium* cp genome was 37.66% and the corresponding values in LSC, SSC and IR regions were 35.71%, 31.55%, 42.92%, respectively.

To explore the phylogenetic position of *P. cirrhifolium*, the cp genomes of 15 representatives of Asphodelaceae was downloaded from NCBI GenBank. The sequences of 78 common protein-coding genes were used for phylogenetic analysis. Complete chloroplast genome sequence alignment was performed by MAFFT program (Katoh and Standley 2013), and then phylogenetic tree was built by IQ-tree software (Nguyen et al. 2015) with the maximum-likelihood algorithm. The phylogenetic analysis (Figure 1) indicated that *P. cirrhifolium* has a close relationship with *P. kingianum*.

**Figure 1.**
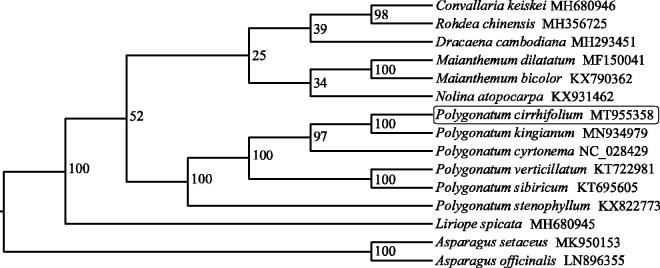
Phylogenetic tree construction using maximum likelihood (ML) based on 78 protein-coding genes from the chloroplast genomes of 15 Asphodelaceae species. The bootstrap support values were shown at the branches.

## Data Availability

The genome sequence data that support the findings of this study are openly available in GenBank of NCBI at (https://www.ncbi.nlm.nih.gov/) under the accession no. MT955358. The associated BioProject, SRA, and Bio-Sample numbers are PRJNA667561, SRR12778006, and SAMN16378112, respectively.
